# Single dose of multi-clade virus-like particle vaccine protects chickens against clade 2.3.2.1 and clade 2.3.4.4 highly pathogenic avian influenza viruses

**DOI:** 10.1038/s41598-021-93060-8

**Published:** 2021-07-02

**Authors:** Yong-Myung Kang, Hyun-Kyu Cho, Ju Hun Kim, Su Jin Lee, Seo-Jeong Park, Do-Young Kim, Seong Yup Kim, Jung-won Park, Myoung-Heon Lee, Min-Chul Kim, Hyun-Mi Kang

**Affiliations:** 1 Avian influenza Research & Diagnostic Division, Animal and Plant Quarantine Agency, 177 Hyeoksin 8-ro, Gimcheon-si, Gyeongsangbuk-do 39660 Republic of Korea; 2 Komipharm Institute, 17 Gyeongje-ro, Siheung-si, Gyeonggi-do 15094 Republic of Korea; 3 Animal Disease Diagnostic Division, Animal and Plant Quarantine Agency, 177 Hyeoksin 8-ro, Gimcheon-si, Gyeongsangbuk-do 39660 Republic of Korea

**Keywords:** Immunology, Microbiology, Diseases

## Abstract

Virus-like particles (VLPs) are recognized as an alternative vaccine platform that provide effective protection against various highly pathogenic avian influenza viruses (HPAIVs). Here, we developed multi-clade VLPs expressing two HAs (a chimera of clade 2.3.2.1c and clade 2.3.4.4c HA) within a single vector. We then compared its protective efficacy with that of a monovalent VLP and evaluated its potency against each homologous strain. Chickens vaccinated with the multi-clade VLP shed less virus and were better protected against challenge than birds receiving monovalent vaccines. Single vaccination with a multi-clade VLP resulted in 100% survival, with no clinical symptoms and high levels of pre-challenge protective immunity (7.6–8.5 log_2_). Moreover, the multi-clade VLP showed high productivity (128–256 HAU) both in the laboratory and on a large scale, making it cheaper than whole inactivated vaccines produced in eggs. However, the PD_50_ (protective dose 50%) of the multi-clade VLP against clades 2.3.2.1c and 2.3.4.4c was < 50 PD_50_ (28 and 42 PD_50_, respectively), and effective antibody response was maintained for 2–3 months. This multi-clade VLP protects against both clades of HPAI viruses and can be produced in high amounts at low cost. Thus, the vaccine has potential as a pandemic preparedness vaccine.

## Introduction

Highly pathogenic avian influenza (HPAI) has been circulating in wild birds and poultry worldwide since the first H5N1 avian influenza virus, A/Goose/Guangdong/1/96, was identified in China in 1996^1,2^. Novel HPAI H5Nx viruses, including various NA, have emerged continuously due to extensive genetic reassortment activity; indeed, various clades have emerged due to genetic evolution and antigenic drift^3–5^. As a result, persistent HPAI outbreaks on poultry farms in many countries have been caused by mutated viruses, resulting in serious economic losses^1^. Moreover, various subtypes of HPAI have emerged simultaneously in countries such as China (H5N6, H5N1), Vietnam (H5N6, H5N1), and Taiwan (H5N2, H5N5), making it difficult to eradicate the virus^6^.

Since the emergence of H5N1 on a poultry farm in 2003, H5 HPAI outbreaks have occurred continuously, and new influenza viruses and genotypes have been introduced into Korea^7,8^. Various clades of H5Nx, including H5N1, H5N6, and H5N8, have been identified^9–13^. In particular, outbreaks of HPAI subtypes H5N6 and H5N8 occurred simultaneously in 2017^14^. During the unprecedented outbreaks of HPAI in 2016/2017, there has been increased demand for AIV vaccination by poultry producers and animal welfare organizations. Potential vaccines in the future should be considered with introduction of two or more viruses simultaneously around Korea.

Vaccination policy may be a supportable measure for preventing HPAI when implemented properly and in conjunction with accurate epidemiologic investigation and control measures^7,15^. Until recently, most AI vaccines used or registered in the field were whole inactivated AI vaccines^16^. VLPs are a vaccine platform that can respond effectively to a wide range of infectious viruses^17,18^. A previous study examined the efficacy of VLP vaccines against multi-clade H5N1^2^, whereas another showed that VLP displaying H5, H7, H9, and N1 protect chicken from infection by heterologous virus^19^. However, preparation of a multivalent vaccine against various HPAI viruses by mixing monovalent whole inactivated vaccines raises biosecurity concerns and is economically unfeasible^20^. Therefore, a vaccine based on VLP requires stronger product development through further studies examining multi-subunit, chimeric, and other types of VLPs; such studies should focus on delivery of sufficient amounts of VLP antigen because these vaccines will not be competitive in the market unless they are both productive and cost-effective^21^.

Here, we constructed a single vector containing cassettes encoding multi-clade VLPs and then manufactured multi-clade VLPs as a vaccine capable of protecting against two separate clades of HPAI virus. We then compared its protective efficacy with that of a monovalent VLP and evaluated its potency against each homologous strain in specific pathogen free (SPF) chicken.

## Results

### Expression and preparation of monovalent and multi-clade VLPs

**Figure 1:**
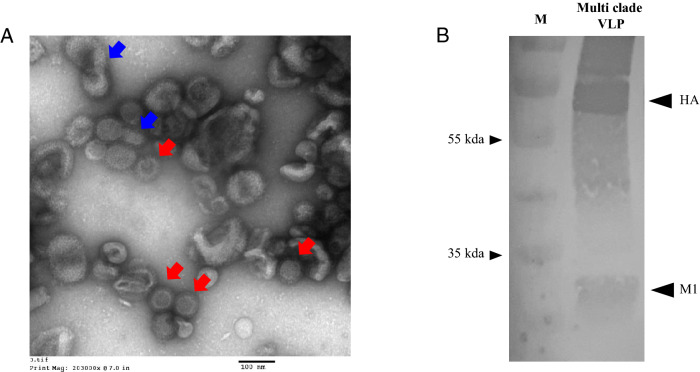
Transmission electron microscopy (EM) and Western blot analysis of multi-clade VLPs. Negative stain (1% uranyl acetate) EM image of multi-clade VLPs (Scale bar is 100 nm, red arrow indicates multi-clade VLP and blue arrow indicate baculoviral particle) (**A**). Blots were incubated with chicken anti-HA antiserum and goat ant-chicken IgG (HRP). HA and M specific bands were detected in preparations of multi clade VLP infected cells (**B**).

Monovalent VLP_ES2, VLP_KA435, and VLP_KA435chi, and multi-clade VLP_ES2/KA435chi vaccines, were produced and secreted successfully from Sf9 cells. The HA titer of VLP_ES2 in the culture supernatant ranged from 128 to 256 HAU. However, the HA titer of VLP_KA435 was as low as 32–64 HAU. The titer of VLP _KA435chi ranged from 128 to 256 HAU. The titer of the multi-clade VLP_ES2/KA435chi was 128–256 HAU. After large-scale production, the titer of the multi-clade VLP was also 128–256 HAU per 25 L of medium (the same as lab-scale production) (Table 1). The multi-clade VLP was purified from the culture supernatant and examined by transmission electron microscopy and western blot (Fig. 1).

**Table 1:** Expression and proliferation of VLPs.

Type of VLP^1^	Cell line	Production system	Volume^2^	HAU^3^
Monovalent VLP ES2	Sf9	Shaker	250 mL	128–256
Monovalent VLP KA435	Sf9	Shaker	250 mL	64
Monovalent VLP KA435chi	Sf9	Shaker	250 mL	128–256
Multi-clade VLP	Sf9	Shaker	250 mL	128–256
Multi-clade VLP (large scale)	Tni	Wave bag reactor	25 L	128–256

*HAU* hemagglutination units, *VLP* virus-like particle.

^1^VLPs were purified from Sf9 or the Tni culture supernatant.

^2^Volume of the cell culture supernatant.

^3^Hemagglutination activity was determined using 1% chicken red blood cells.

### Comparison of the efficacy between monovalent and multi-clade VLP vaccination

Next, we compared the protective efficacy of the monovalent and multi-clade VLP vaccines in SPF chickens infected with KA435/2.3.2.1c and ES2/2.3.4.4c. A 100% survival rate was observed in all groups of SPF chickens, except the group receiving the VLP_KA435 vaccine (90%), after challenge with homologous HPAI (Table 2). All SPF chickens vaccinated with VLPs had seroconverted before challenge, with a mean HI titer of 5.6–9.0 log_2_ against each antigen. SPF chickens vaccinated with the multi-clade VLP had a mean HI titer of 7.0–7.3 log_2_ against both antigens. In SN test, sera from the multi-clade VLP vaccinated chickens induced mean SN titer 72–524 (range from 20 to 1280) against both antigens before challenge, and sera from monovalent VLP vaccinated chickens induced mean SN titer 26 and 62 (range from 0 to 160) against each homologous antigen although they show no reaction in cross SN test. After challenge with KA435/2.3.2.1c and ES2/2.3.4.4c, all vaccinated chickens produced green stools and diarrhea, and some became lethargic. Virus shedding was detected from 1–5 dpi, with a viral titer of 10^1.3^–10^2.2^ TCID_50_/0.1 mL in OP swab samples and 10^1.6^–10^2.0^ TCID_50_/0.1 mL from 1 to 5 dpi in CL swab samples. In the multi-clade VLP vaccinated groups, virus shedding was detected at 1 dpi in an OP sample from one chicken, with viral titer of 10^1.3^ TCID_50_/0.1 mL, after challenge with KA435/2.3.2.1c; no virus shedding was detected in birds challenged with ES2/2.3.4.4c. In the sham groups challenged with KA435/2.3.2.1c and ES2/2.3.4.4c, virus shedding was detected at 1 dpi in seven chickens, with a viral titer of 10^2.0^–10^2.2^ TCID_50_/0.1 mL only in the OP swab sample. There were significant differences (*p* < *0.05*) at 1 dpi in the viral titers in OP swab samples from sham-vaccinated chickens and SPF chickens vaccinated with the multi-clade VLP vaccine against KA435/2.3.2.1c (Table 2).

**Table 2:** Results among single and multivalent vaccinations against homologous and heterologous HPAI.

Vaccine strain	Antigen dose (HAU)	HI titer(log_2_) at 3 wpv^1^		SN titer at 3 wpv^2^		Challenge Strain^3^	Morbidity	Survival (%) (MDT)^4^	Virus shedding (TCID_50_/0.1 ml)^5^					
		KA435	ES2	KA435	ES2				1dpi		3dpi		5dpi	
									OP	CL	OP	CL	OP	CL
VLP/KA435chi	512	10/10 (9.0)	8/10 (1.9)	5/10 (26)	0/10 (–)	KA435 /2.3.2.1c	Lethargy, Diarrhea	90 (7)	0/10 (–)	0/10 (–)	1/10 (1.3)	1/10 (2.0)	2/10 (1.4 ± 0.1)	2/10 (1.6 ± 0.4)
Multi-clade VLP		10/10 (7.0)	10/10 (7.8)	10/10 (76)	10/10 (416)		diarrhea	100 (–)	1/10* (1.3)	0/10 (–)	0/10 (–)	0/10 (–)	0/10 (–)	0/10 (–)
Sham	–	0/10 (–)	0/10 (–)	0/10 (–)	0/10 (–)		Lethargy, death	0 (2.4)	7/10 (2.2 ± 0.8)	0/10 (–)	NT	NT	NT	NT
VLP/ES2	512	8/10 (1.7)	10/10 (5.6)	0/10 (–)	10/10 (62)	ES2 /2.3.4.4c	Diarrhea,	100 (–)	0/10 (–)	0/10 (–)	1/10 (1.3)	0/10 (–)	2/10 (1.4 ± 0.2)	1/10 (2.0)
Multi-clade VLP		10/10 (7.1)	10/10 (7.3)	10/10 (72)	10/10 (524)		Diarrhea,	100 (–)	0/10 (–)	0/10 (–)	0/10 (–)	0/10 (–)	0/10 (–)	0/10 (–)
Sham	–	0/10 (–)	10/10 (–)	0/10 (–)	0/10 (–)		Lethargy, death	0 (2.5)	7/10 (2.0 ± 0.7)	0/10 (–)	NT	NT	NT	NT

*HA* hemagglutination activity, *NT* not tested, *dpi* days post-infection, *OP* oropharyngeal, *CL* cloacal, *HI* hemagglutination inhibition.

*p value < *0.05.*

^1^No. serology positive/total survived in group (mean HI titer).

^2^No. serology positive/total survived in groups (mean SN titer).

^3^Homologous: same as vaccine strain; heterologous: different from vaccine strain.

^4^MDT = mean death time (days).

^5^No. virus positive/total in group (mean shed-virus titer).

### Potency and efficacy of the chimeric multi-clade VLP vaccination against each homologous challenge

#### Clinical protection

**Figure 2:**
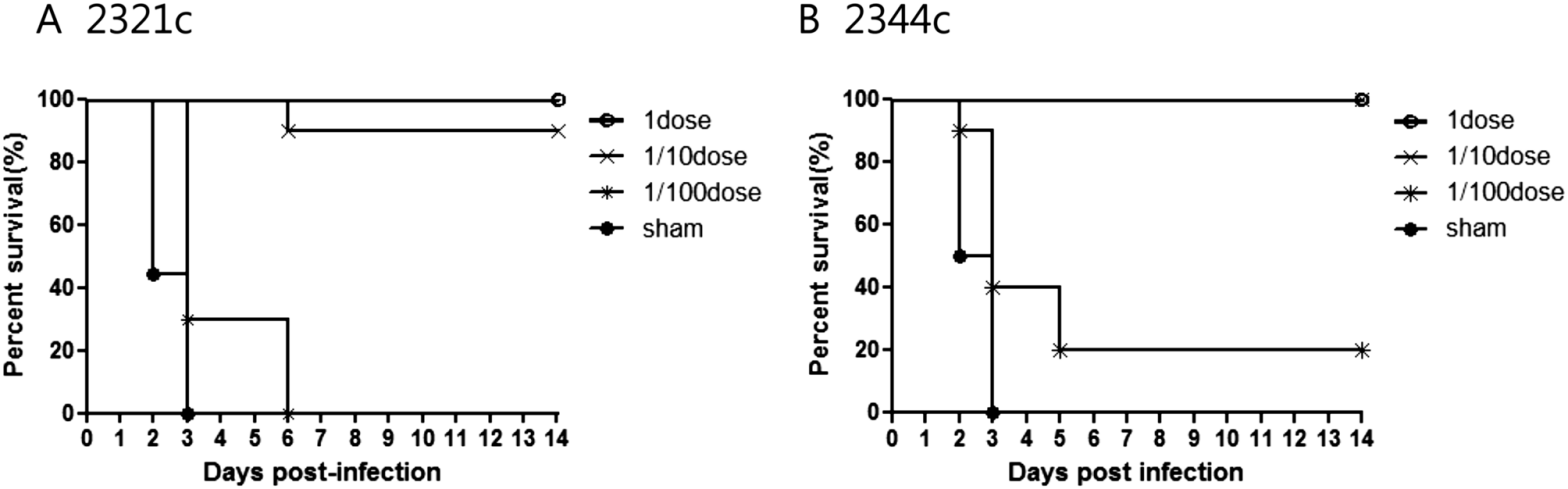
Survival of vaccinated chickens challenged with each homologous HPAIV. Survival of sham-vaccinated chickens and chickens inoculated with one (1) dose, one-tenth (1/10) dose, or one-hundredth (1/100) dose of the multi-clade VLP and then challenged with each homologous H5 HPAIV: (**A**) KA435/2.3.2.1c. (**B**) ES2/2.3.4.4c.

For SPF chickens, vaccination with one dose conferred 100% clinical protection, with no clinical symptoms evident after challenge with KA435/2.3.2.1c and ES2/2.3.4.4c. However, the group vaccinated with one-tenth dose and then challenged with KA435/2.3.2.1c suffered 20% mortality by 6 dpi (Fig. 2A). Vaccination with one-hundredth dose resulted in even higher mortality (around 70%) at 6 dpi. Mortality in the group receiving one-hundredth dose of the multi-clade VLP vaccine and then challenged with KA435/2.3.2.1c was 100%, showing neurological signs and diarrhea; however, in the group challenged with ES2/2.3.4.4c, eight chickens died at 7–8 dpi post-challenge (Fig. 2B) after showing neurological signs and diarrhea. The mean time to death in one-hundredth dose vaccination groups challenged with either virus was 3.3–3.4 days. For sham-treated chickens, the mean time to death was 2.5–2.6 days.

Clinical protection was also indicated by the vaccine potency (PD_50_) results^22^, with SPF chickens challenged with the KA435 or ES2 viruses showing potency values of 28 and 42 PD_50_, respectively (Table 3).

**Table 3:** Results of vaccination with varying doses of multi-clade VLP against two homologous HPAI.

Vaccine	Antigen dose^1^	Challenge strain	Survival (%) (MDT)^2^	Peak shedding (3 dpi)^3^		HI titer(log_2_)^4^ against challenge antigen		PD_50_
				OP	CL	Pre-challenge	Post-challenge	
Multi-clade VLP	1	KA435	100 (–)	2/10 (1.8)	0/10 (–)	10/10(7.2)	10/10(7.9)	28
	1/10	KA435	90 (6)	2/10 (3.3)	2/10 (3.9)	10/10(3.8)	9/9(6.4)	
	1/100	KA435	0 (3.9)	3/3 (2.6)	3/3 (3.0)	6/10(1.0)	NT	
	sham	KA435	0 (2.4)	NT	NT	0/10(–)	NT	
	1	ES2	100 (–)	0/10 (–)	0/10 (–)	10/10(7.8)	10/10(8.5)	42
	1/10	ES2	100 (–)	3/10 (1.4)	1/10 (4.3)	10/10(4.7)	10/10(7.6)	
	1/100	ES2	20 (3.4)	3/4 (2.2)	2/4 (4.0)	2/10(1.0)	2/2(6.0)	
	sham	ES2	0 (2.5)	NT	NT	0/10(–)	NT	

*HA* hemagglutination activity, *NT* not tested, *dpi* days post-infection, *OP* oropharyngeal, *CL* cloacal, *HI* hemagglutination inhibition, *PD*_*50*_ protective dose 50%

*p value < *0.05.*

^1^One dose (1) contained 512 HAU (hemagglutination units).

^2^MDT, mean death time (days).

^3^No. of virus positive/total in the group (mean shed-virus titer).

^4^No. of serology positive/total surviving in the group (mean HI titer).

#### Serology

**Figure 3:**
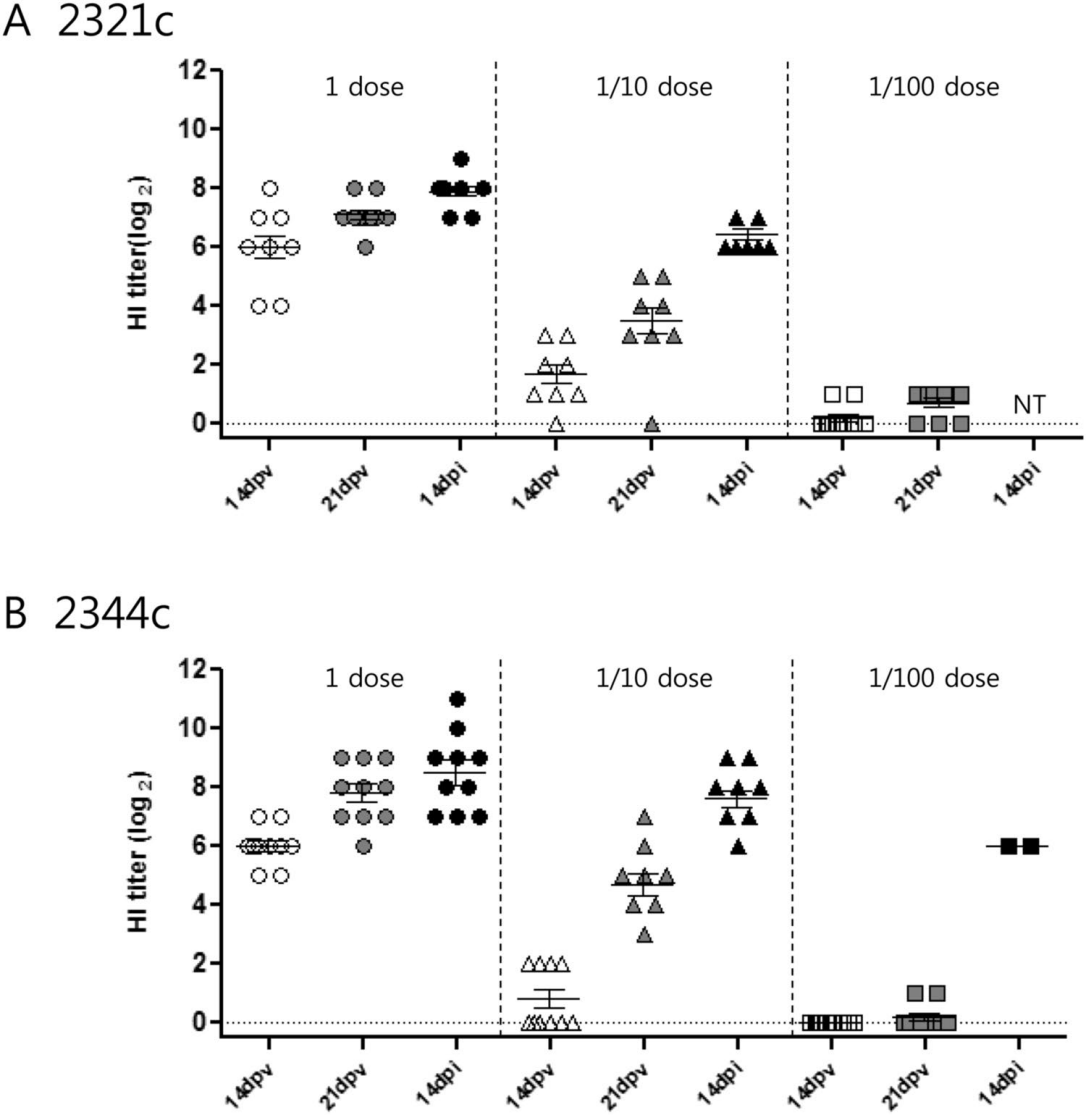
Serological responses in vaccinated and challenged chickens. Hemagglutination inhibition (HI) assay titers in vaccinated chickens measured at different times post-vaccination and challenged with homologous virus. HI titers were assessed at 14 days post-vaccination (dpv), at 21 dpv, and at 14 days post-infection (dpi) with homologous virus. Chickens received one (1) dose, one-tenth (1/10) dose, or one-hundredth (1/100) dose. (**A**) Challenge with KA435/2.3.2.1c. (**B**) Challenge with ES2/2.3.4.4c. Individual data points are shown, along with mean and standard error.

All vaccinated groups showed detectable antibody titers against ES2 and KA435 antigens pre-challenge and post-challenge; these titers increased over time (Fig. 3). In vaccinated SPF chickens (one dose or one-tenth dose), all those receiving the multi-clade VLP vaccine seroconverted before challenge, with a mean tier of 7.2–7.8 and 3.9–4.7 log_2_ after one dose and one-tenth dose, respectively. After challenge, the antibody titer against ES2 and KA435 antigens increased to 7.9–8.5 log_2_ and 6.5–7.6 log_2_ in the one dose and one-tenth dose groups, respectively. Unlike the one dose- and one-tenth dose-vaccinated groups, the one-hundredth dose-vaccinated groups showed a weaker antibody response (1.0 log_2_) prior to challenge. Following challenge, the HI titer of surviving chickens challenged with ES2 had more various antibody responses, with HI titers around 6.0 log_2_ (Fig. 3B). None of the sham-vaccinated chickens had detectable HI antibodies before challenge (data not shown).

#### Virus shedding

**Figure 4:**
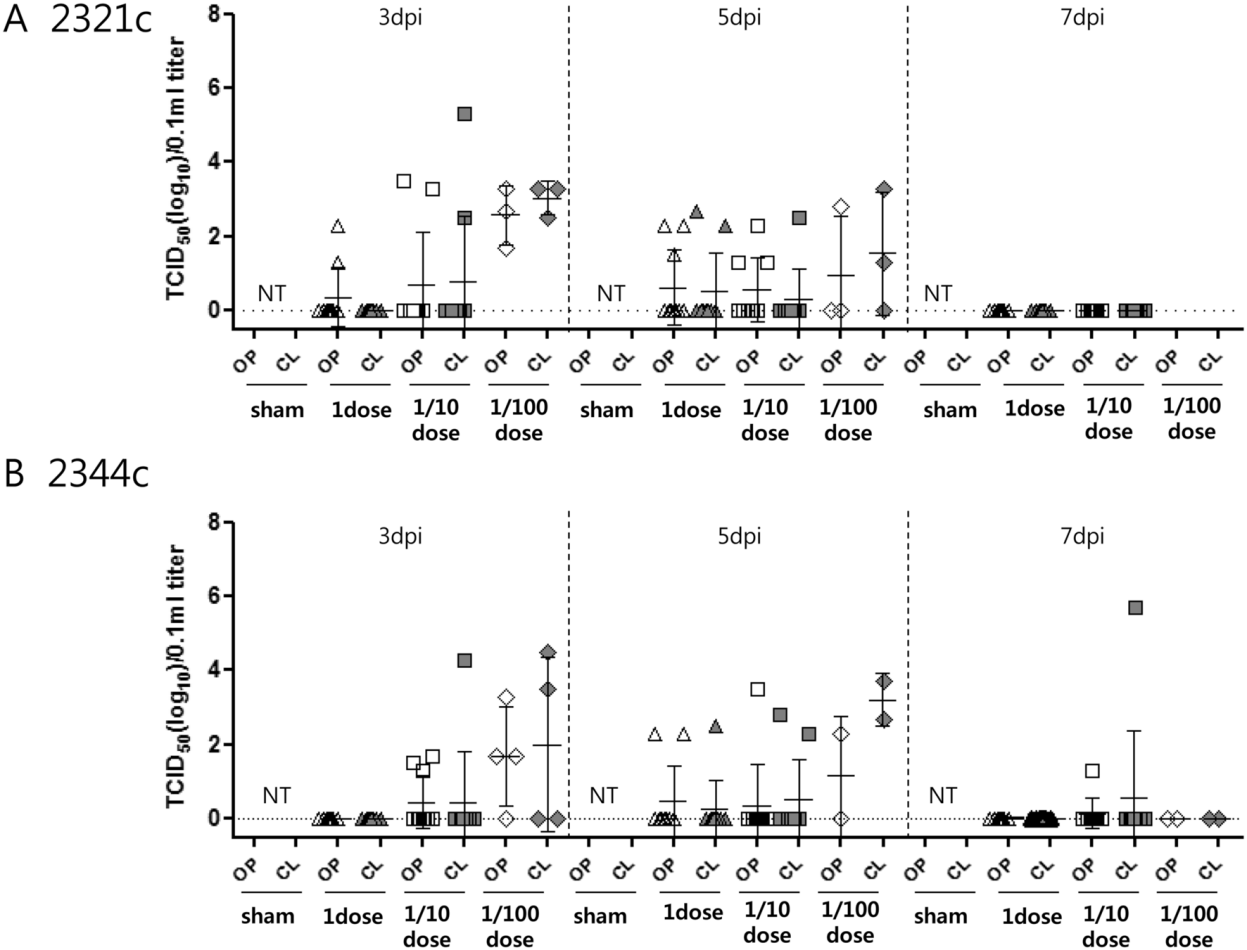
Virus shedding in oropharyngeal (OP) and cloacal (CL) swab samples after inoculation with homologous HPAIV. Titers of virus shed in OP and CL samples from chicken sham-vaccinated or inoculated with vaccines at one (1) dose, one-tenth (1/10) dose, or one-hundredth (1/100) dose; titers were measured at 3, 5, and 7 days post-infection (dpi) with each homologous H5 HPAIV. Viral titers are expressed as log_10_TCID_50_ (50% tissue culture infectious dose)/0.1 mL, with error bars. (**A**) KA435/2.3.2.1c. (**B**) ES2/2.3.4.4c. The lower limit of detection was 1 log_10_TCID_50_/0.1 mL.

As shown in Fig. 4, little virus shedding was observed from 3 to 7 dpi in SPF chickens vaccinated with one dose of the multi-clade VLP. However, virus shedding was detected from 3 to 7 dpi in SPF chickens vaccinated with one-tenth dose of the multi-clade VLP and challenged with either KA435/2.3.2.1c or ES2/2.3.4.4c. In SPF chickens vaccinated with one-hundredth dose, virus shedding was detected in surviving birds, with a viral titer of 10^1.7^–10^2.8^ TCID_50_/0.1 mL in OP swab samples from 3–5 dpi and 10^2.4^–10^4.0^ TCID_50_/0.1 mL in CL swab samples from 3 to 5 dpi. Most birds in the sham-vaccinated groups were not tested because they died prior to sampling (Fig. 4).

### Antibody persistence

**Figure 5:**
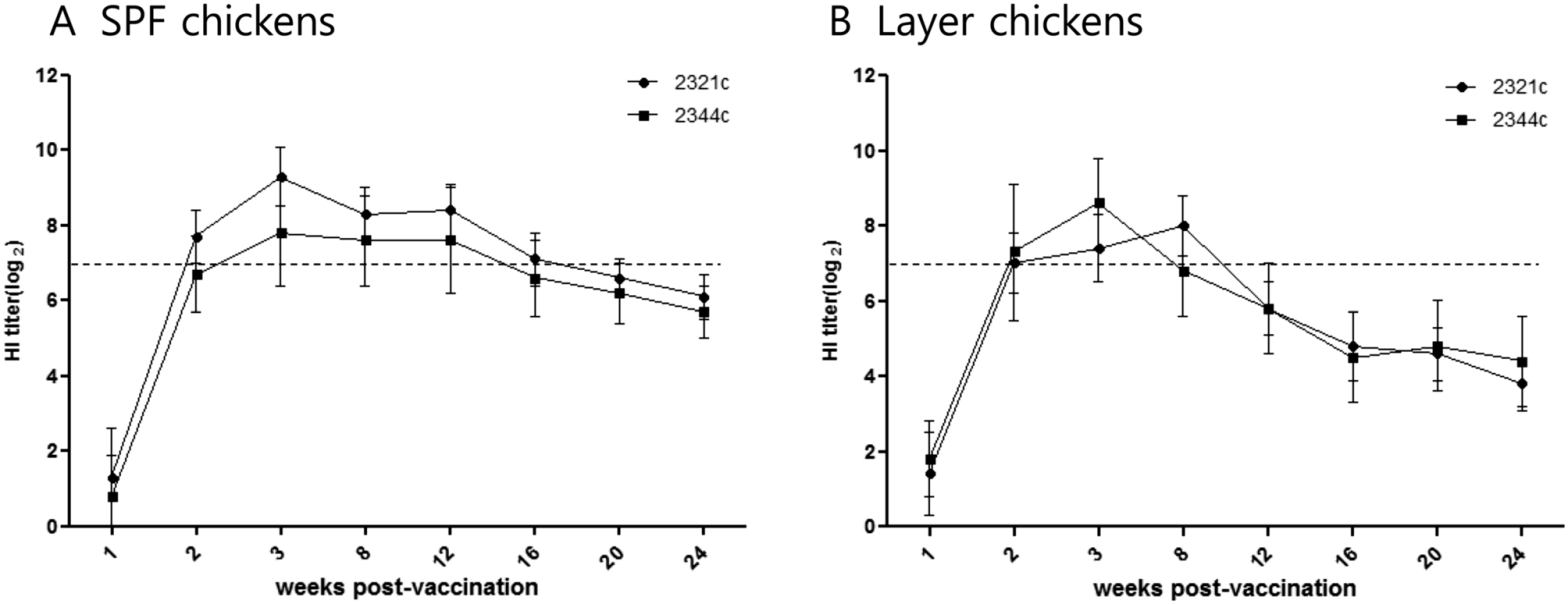
Antibody persistence following administration of one dose (512 HAU) of vaccine. (**A**) SPF chickens. (**B**) Layer chickens. Hemagglutination inhibition (HI) assay titers were determined up to 24 weeks post-vaccination. Titers are expressed as log_2_ values. The horizontal dotted line indicates a HI titer of 7 log_2_, which is the standard threshold for preventing virus shedding.

We measured the mean HI titers against ES2 and KA435 antigens in SPF chickens and layer chickens inoculated with one dose of the multi-clade VLP vaccine. In SPF chickens vaccinated with the multi-clade VLP, HI titers against the KA435 antigen were 9.3 log_2_ and those against the ES2 antigen were 7.8 log_2_ at 3 wpv. The mean HI titers peaked between 3 wpv, and remained above 7 log_2_ to provide reduction in challenge virus replication and shedding^23^ by 12 wpv (KA435) and 16 wpv (ES2) (Fig. 5A). In layer chickens vaccinated with the multi-clade VLP, HI titers against the KA435 antigen were 7.4 log_2_, and those against the ES2 antigen were 8.6 log_2_ at 3 wpv. The mean HI titers peaked at 3 wpv, and remained above 7 log_2_ by 8 wpv (KA435) and 3 wpv (ES2). None of the chickens showed HI titers above 7 log_2_ at 24 wpv (Fig. 5B).

## Discussion

Here, we constructed a vector containing cassettes for multi-clade VLP into which two kinds of HA can be inserted. We then manufactured a multi-clade VLP vaccine by inserting the HA sequence of clade 2.3.2.1c(chimera) and 2.3.4.4c vaccine strains from the Korean AI national antigen bank. Finally, we compared the efficacy of monovalent and multi-clade VLP vaccines in SPF chickens challenged with homologous strains.

Chickens vaccinated with the multi-clade VLP shed less virus and were better protected against challenge than chickens vaccinated with the monovalent vaccine. This is likely because multivalent vaccines trigger stronger cellular and humoral responses, thereby inhibiting viral replication more effectively than the monovalent VLP vaccines^24,25^. Moreover, one chicken vaccinated with the monovalent VLP_KA435chi died, despite showing a higher HI titer (9.0 log_2_) (Table 2); this may be because the HA2 of KA435 was substituted with the HA2 of Buan2, coupled with challenge with the more virulent 2.3.2.1c strain as reported previously^2,6^, and induction of lower SN titer which more closely related to the capability of antibodies controlling the replication of virus comparing HI titer^27^.

For use in an antigen bank, an emergency vaccine should show a minimum 50 PD_50_ per dose, or antibody persistence above and HI of 1/128 HI for 6 months post-vaccination; in Korea, the guidelines are a minimum 80% protection from mortality and an HI titer of greater than 1/128^14^. In this study, the PD_50_ and antibody persistence of the multi-clade VLP did not meet these criteria or were lower than those of whole inactivated vaccinated chickens in comparison with the results of a previous study^2,6^. Whole inactivated vaccines induce a stronger immune response than subunit vaccines such as VLP vaccines^28^. Therefore, we believe that our results are due to the fact that the VLP vaccine is based on part (M and HA) of the virus. Indeed, other studies show that whole inactivated vaccines are more effective in potency tests than split, subunit vaccines and virosomes; also, whole inactivated vaccines have a significantly higher probability of inducing seroconversion than subunit vaccines^29,30^. Further study is needed on how multi-clade VLP increases protective efficacy comparing to whole inactivated vaccines.

Our multi-subtype vector includes a cassette into which two full HAs are co-localized within one VLP structure^2^; we then ensured high protein expression (128–256 HAU) by producing a chimeric VLP under optimized cell culture conditions. Indeed, productivity in cell culture was equal to that after large-scale (25 L) production (Table 2), and higher than that after large-scale production reported by other studies (e.g., 16–128 HAU in Sf9 cells)^2,31^. This process enables cost savings of approximately 0.02 dollars per dose when compared with mixing two types (0.068 dollars per dose) of whole inactivated vaccine using the egg product system of the Korean AI national bank (data not shown). This means that the multi-clade VLP we developed in this study may be economically more viable than vaccines cultured in eggs. In addition, multi-clade VLPs effectively induce Th1 type immune responses, and plasma and memory B cells^32^. Only single vaccination of multi-clade VLP with a dose of 512 HAU showed immunogenicity, survival rate and reduction of virus shedding in similar to results boosting other VLP vaccination^2,19,33^.

In conclusion, we constructed a single vector containing cassettes encoding multi-clade VLPs and produced a chimeric vaccine capable of protecting chickens against simultaneous challenge with clade 2.3.2.1c and 2.3.4.4c HPAIVs. Large amounts of vaccine were produced in cell culture, making the vaccine potentially cost-effective. However, the PD_50_ and antibody persistence were below national standards and lower than those of whole inactivated vaccines. Taken together, these results suggest that the multi-clade VLP vaccine is an economically effective vaccine that protects against infection by multiple HPAIVs.

## Materials and methods

### Cell and viruses

Sf9 insect cells (Gibco, Waltham, MA, USA) and Trichoplusia ni (Tni) insect cells were maintained in suspension in SF-900 III SFM medium (Gibco) and ESF 921 (Expression system, Davis, CA, USA), respectively, at 27 °C. Three different H5 HPAIVs from the Korean national antigen bank^14^ were used as seed strains and challenge strains. A/duck/Korea/ES2/2016(H5N6, clade 2.3.4.4c)^12^, hereafter called ES2/2.3.4.4c, and A/broiler duck/Korea/Buan2/2014(H5N8, clade 2.3.4.4a)^11^, hereafter called Buan2/2.3.4.4a, were isolated from poultry farms in Korea; A/chicken/Vietnam/NCVD-KA435/13(H5N1, clade 2.3.2.1c)^14^, hereafter called KA435/2.3.2.1c, was kindly provided by the National Center for Veterinary Diagnostics in Vietnam. A/Puerto Rico/8/34 (H1N1), hereafter named PR8, was used for cloning of the M1 gene. Viruses were propagated for 60 h in 10-day-old embryonated eggs from SPF chickens.

### Cloning of HA and M genes

Viral RNA was extracted from ES2/2.3.4.4c, KA435/2.3.2.1c, Buan2/2.3.4.4a, and PR8 using RNeasy mini kit (Intron, Korea). To amplify DNA from extracted viral RNA, polymerase chain reaction (PCR) was performed using the RT kit (Intron) and specific primers containing restriction enzyme sequences. The following primer pairs were used for PCR amplification of modified HA genes with deletion of the polybasic amino acid region and the M1 gene: HA_ES2 (F) 5′-CGTGCGGGATCCATGGAGAAAATAGTGCTTCT-3′ and (R) 5′-AATAGGAAGCTTTTAAATGCAAATTCTTGCATT-3′; HA_KA435 (F) TGGACTACTAGTATGGAGAAGATCGTTCTTCT-3′ and (R) 5′-GAGTACGTCGACTTAAATGCAAATACGCACT-3′; M1_PR8 (F) CAATCGAGCATGCTCTCCCTCTTGAGCTTCCTA-3′ and (R) 5′-TGCCAGTCCCGGGATGAGTCTTCTAACCGAGGT-3′; HA1_KA435 (F) 5′-TGGACTACTAGTATGGAGAAGATCGTTCTTCT-3′ and (R) 5′-AGGAGTGGCGCCCCAAACAGTCCTCTTTTGCG-3′; HA2_Buan2 (F) 5′-TGTAAGCCTAGGATGGAGATAATTAAAATGAT-3′ and (R) 5′-AGCGGTCCTAGGATCAGATCCAGACATGATAA-3′. Amplified HA and M1 genes were cloned into the TA cloning vector pGEM®-T (Promega, Madison, WI, USA), and each sequence was confirmed by DNA sequencing.

### Construction of expression vectors and generation of recombinant baculovirus

**Figure 6:**
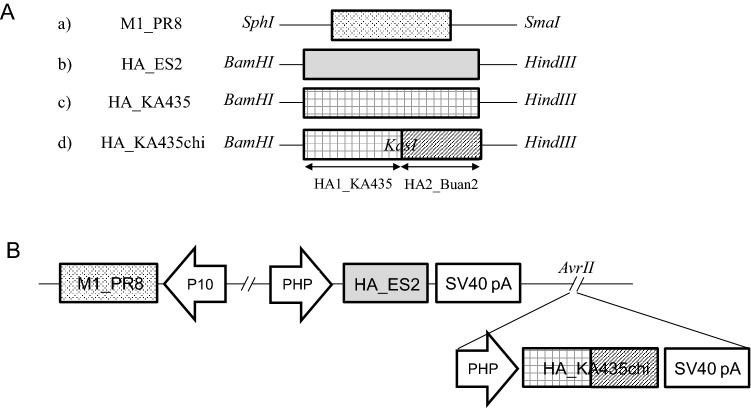
Baculovirus construct for production of multi-clade VLPs. (**A**) (**a**) M1 of PR8 (M1_PR8) is inserted between restriction enzyme sites for SphI and SmaI, and the HAs of (**b**) ES2, (**c**) KA435 (HA_ES2 and HA_KA435), and (**d**) chimera of KA435 (HA_KA435chi) are inserted between restriction sites for BamHI and HindIII. (**B**) The M1 gene is under the control of the p10 promoter (P_P10_); the HA of ES2 (HA_ES2) is under the control of the polyhedron promoter (P_PH_); and the HA chimera of KA435 (HA_KA435chi), inserted into the AvrII restriction site, is under the control of the polyhedron promoter (P_PH_).

M1 of PR8 (M1_PR8) digested with SmaI/SphI and T4 DNA ligase (Takara, Japan) was used to ligate the resulting M1 DNA fragment into the SmaI/SphI-digested multi-cloning site (MCS) pFastBac™ dual vector (Invitrogen, Carlsbad, CA, USA) under the control of promotor10 (p10) (Fig. 6Aa). HA of ES2 (HA_ES2) and HA of KA435 (HA_KA435) were digested by BanH1/HindIII, and the resulting HA DNA fragments were each ligated into the BanH1/HindIII-digested polyhedron (PH) of the MCS pFastBac™ dual vector (Invitrogen) (Fig. 6Ab,Ac). To increase protein expression, HA_KA435 was constructed as a chimeric structure (HA_KA435chi). HA2 of KA435 and HA2 of Buan2 (HA_Buan2) were digested by KasI/HindIII, and HA2 of HA_KA435 was substituted with HA2 of HA_Buan2 (Fig. 6Ad). HA_KA435chi was digested by AvrII, and the resulting HA DNA fragment was ligated into the AvrII site of the pFastBac™ dual vector to generate a multi-clade VLP containing M1_PR8, HA_ES2, and HA_KA435chi (HA_ES2/KA435chi) (Fig. 6B).

The constructed pFastBac™ dual vector plasmids pHA_ES2, pHA_KA435, pHA_KA435chi, and pHA_ES2/KA435chi were transformed into DH10Bac™ competent cells (Invitrogen) to generate recombinant bacmids. Recombinant bacmid DNA was transfected into Sf9 cells seeded in 6-well plates (8 × 10^5^ cells/well) using Cellfectin® Reagent (Invitrogen), resulting in the release of recombinant baculovirus (rBV) into the culture medium. At 72 h post-transfection, culture medium was harvested and inoculated into Sf9 cells to generate a high titer rBV stock. Titration of the baculovirus stock was performed in a plaque assay on Sf9 cells.

### Vaccine development

To express each monovalent VLP (VLP_ES2, VLP_KA435, and VLP_KA435chi) and multi-clade VLP (HA_ES2/KA435chi), Sf9 cells (2 × 10^6^ cells/mL) were infected for 3 days with recombinant baculovirus expressing HA and M1 (for monovalent HA expression), or with two HAs and M1 (for multi-clade protein expression) at a multiplicity of infection of 1. The cultured medium containing VLPs was collected and centrifuged for 10 min at 2000 × *g* to remove cell debris. The culture supernatant was chemically treated with formalin (final concentration, 0.1%) to inactivate baculovirus. For large-scale production, Tni insect cells^3,4^ under optimized condition in similar with that of Sf9 cells were infected with recombinant baculovirus expressing VLPs. VLPs were harvested from the medium supernatant, purified on sucrose gradient and concentrated by pump system (100 kDa filtration) (Masterflex). VLP expression of influenza protein was confirmed by Coomassie staining of sodium dodecyl sulfate polyacrylamide gels, western blot carried out using chicken anti-HA antiserum and goat anti chicken IgG (HRP)(KPL) and by nucleotide sequence analysis and hemagglutinin inhibition (HI) tests against homologous antibody. Viral growth was tested in a hemagglutination activity (HA) assay, and titers were adjusted to yield 512 HA units per dose after emulsifying (30:70, w/w) in Montanide ISA VG70 oil adjuvant (SEPPIC, La Garenne-Colombes, France)^3,5^.

### Transmission electron microscopy of VLPs

The VLP suspension was placed on formvar-coated (copper 300 mash) grids, negatively stained with 1% uranyl acetate, and dried by aspiration. The VLP particles were then examined under a transmission electron microscope (Hitachi7100FA, Tokyo, Japan).

### Efficacy of the monovalent and multi-clade VLP vaccines

To compare the efficacy of the monovalent vaccines with that of the multi-clade VLP vaccine, 60 5-week-old SPF chickens were divided into six groups (10 chickens per group). The four groups used for vaccination comprised two groups of chickens receiving monovalent VLP vaccines (VLP_ES2 or VLP_KA435chi) and two groups receiving the multi-clade VLP (VLP_ES2/KA435chi) vaccine. All VLP vaccines were emulsified in Montanide ISA VG70 adjuvant and injected via the intramuscular route; each chicken received 0.5 mL. Another 20 SPF chickens were split into two sham groups and injected with an emulsified solution of PBS plus ISA VG70 in the same ratio as the VLP vaccines. Serum samples were collected from all chickens at weekly intervals post-vaccination and then at 14 days post infection (dpi). To determine the immunogenicity of the VLP vaccines, all sera were tested by HI test against homologous and heterologous, and Serum neutralizing antibody test (SN test) in Dermal Fibroblast 1 (DF1) cells, as described previously^36^. Three weeks after vaccination, two groups of vaccinated chickens were challenged with 0.1 mL ES2/2.3.4.4c and KA435/2.3.2.1c (10^6.0^ EID_50_/0.1 mL). All birds were observed daily for 14 dpi to check mortality, clinical signs, and viral shedding. To determine viral shedding, oropharyngeal (OP) and cloacal (CL) swab samples were collected at 1, 3, 5, 7, 10, and 14 dpi. Each OP or CL sample was suspended in 1 mL of maintenance medium containing an antibiotic–antimycotic mixture (Invitrogen). Samples were used to inoculate cultures of Dermal Fibroblast 1 (DF1) cells, and virus growth was determined by detection of cytopathic effects and measurement of HA activity. Virus titers were calculated as described elsewhere^22^. The limit of virus detection was < 1 log_10_ TCID_50_/0.1 mL. All experiments with live H5 virus were performed in biosafety level 3 facilities and in accordance with guidelines approved by the Animal Ethics Committee of the Animal and Plant Quarantine Agency, Korea (approval number: 2019-492 and 2020-544). Our study is conducted in accordance with ARRIVE guidelines (https://arriveguidelines.org).

### Potency and efficacy of multi-clade VLP vaccination against each homologous challenge

To evaluate the potency (PD_50_, protective dose 50%) and efficacy of the multi-clade VLP vaccine, 40 6-week-old SPF chickens for each HA antigen (ES2/2.3.4.4c and KA435/2.3.2.1c) were divided into four groups (10 chickens per group): three immunization groups and one non-immunization (sham) group. Chickens were immunized intramuscularly with one dose, one-tenth (1/10) dose, or one-hundredth 1(1/100) dose of vaccine in Montanide ISA VG70. The sham group was inoculated with PBS in ISA VG70. At 3 weeks post-vaccination (wpv), chickens were challenged intranasally with 10^6^ EID_50_ (in 0.1 mL) of a virus homologous to the vaccine strain. Post-challenge, chickens were monitored daily for clinical signs and mortality. The PD_50_ was calculated using mortality as the end point, as described previously^22^.

#### Serology and antibody assays

To determine the immunogenicity of the VLP vaccines, serum samples were collected from all chickens prior to vaccination and then at weekly intervals post-vaccination. Serum samples were also obtained from all living chickens at 14 days post-challenge. All sera were subjected to HI tests. The HI titer against clade 2.3.2.1c and clade 2.3.4.4c antigens was measured using OIE standard HI methods.

#### Post-challenge virus shedding

OP and CL swabs were collected from animals in all groups at 3, 5, 7, 10, and 14 dpi. Each sample was suspended in 1 mL of maintenance medium containing an antibiotic–antimycotic mixture (Invitrogen). Samples were treated and inoculated onto DF1 cells as mentioned above.

### Antibody persistence

To determine the persistence of protection provided by the VLP vaccines, 10 6-week-old SPF chickens and 10 14-week-old unimmunized layer chickens (these chickens did not even have the H9N2 vaccine) from a poultry farm in Korea were immunized with a single dose. Blood samples were collected from all immunized chickens every month for 6 months post-vaccination.

### Statistical analysis

Data were analyzed using Prism version 5.0 software (GraphPad Software, Inc., La Jolla, CA, USA). Comparison of serum titers between the groups was made using one-way analysis of variance (ANOVA). Survival rates among groups were analyzed using the log-rank test. The statistical significance of differences between measurements was determined using Student’s *t*-test. A *p*-value < 0.05 was deemed significant.

### Ethical statements

All experiments with live H5 virus were performed in biosafety level 3 facilities and in accordance with guidelines approved by the Animal Ethics Committee of the Animal and Plant Quarantine Agency, Korea (approval number: 2019-492 and 2020-544). Our study is conducted in accordance with ARRIVE guidelines (https://arriveguidelines.org).

## Supplementary Information

Supplementary Information 1.

Supplementary Information 2.

Supplementary Information 3.
