# Supplementary figures and images for: Single dose of multi-clade virus-like particle vaccine protects chickens against clade 2.3.2.1 and clade 2.3.4.4 highly pathogenic avian influenza viruses
Source: Sci Rep. 2021 Jul 2;11:13786. doi: 10.1038/s41598-021-93060-8 (PMC8253753; doi:10.1038/s41598-021-93060-8)

## Slide 1
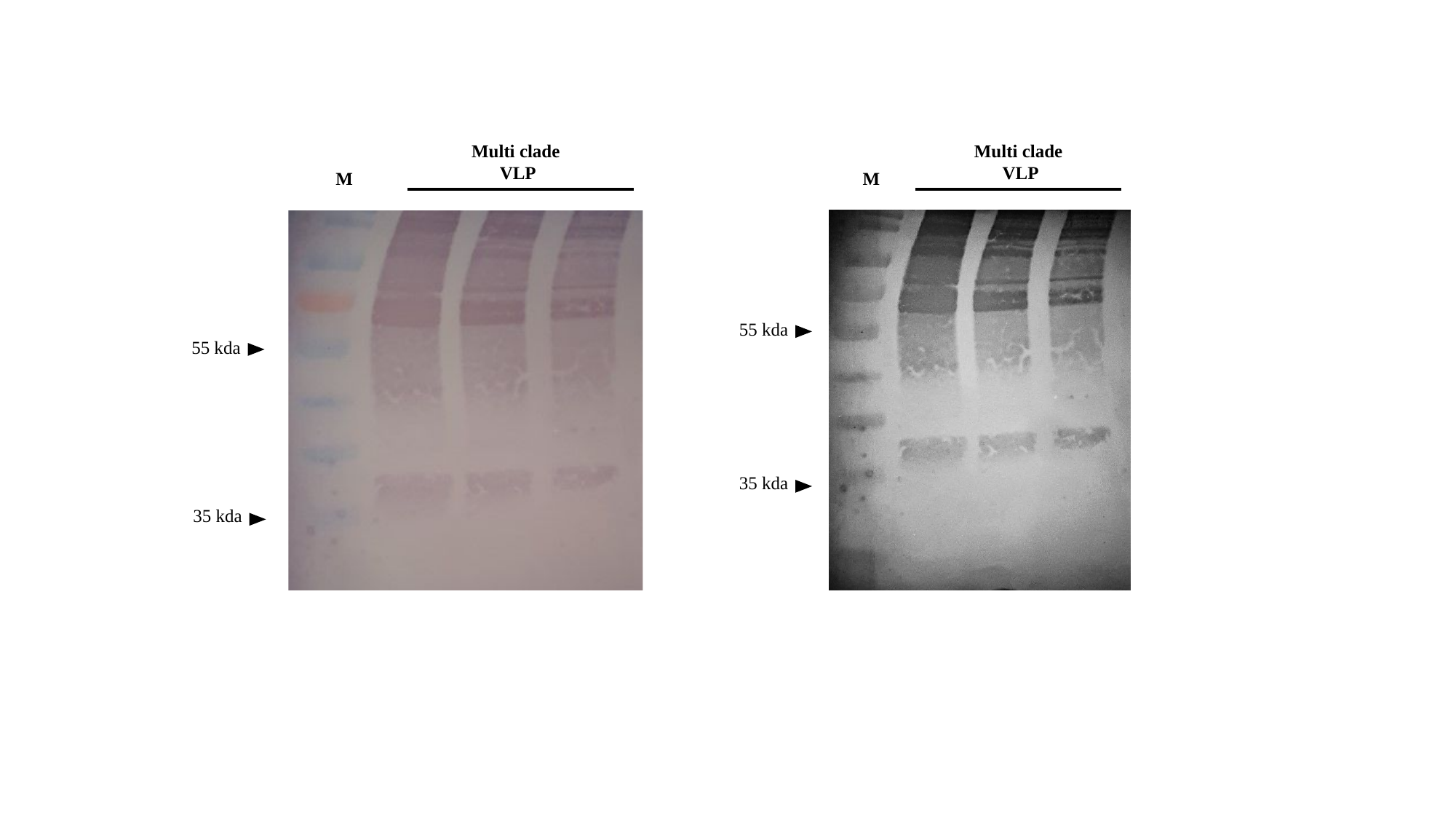

Multi clade
 VLP
Multi clade
 VLP
M
M
55 kda
55 kda
35 kda
35 kda

Supplement: Supplementary file 1 — Supplementary Information 1. [file 41598_2021_93060_MOESM1_ESM.pptx]

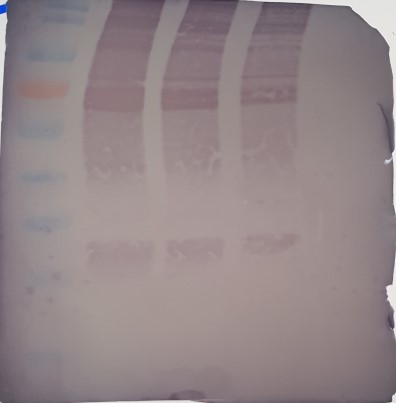

Supplement: Supplementary file 2 — Supplementary Information 2. [file 41598_2021_93060_MOESM2_ESM.jpg]

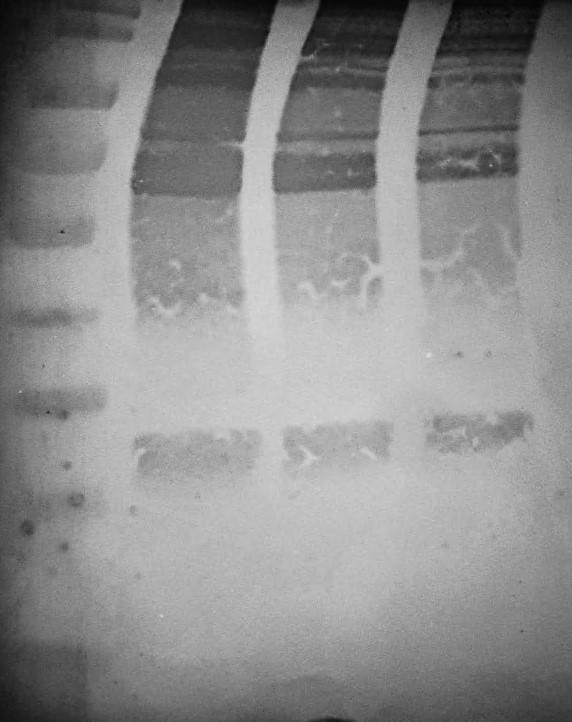

Supplement: Supplementary file 3 — Supplementary Information 3. [file 41598_2021_93060_MOESM3_ESM.jpg]
